# Fenobody and RANbody-based sandwich enzyme-linked immunosorbent assay to detect Newcastle disease virus

**DOI:** 10.1186/s12951-020-00598-2

**Published:** 2020-03-14

**Authors:** Pinpin Ji, Jiahong Zhu, Xiaoxuan Li, Wenqi Fan, Qianqian Liu, Kun Wang, Jiakai Zhao, Yani Sun, Baoyuan Liu, En-Min Zhou, Qin Zhao

**Affiliations:** 1 Department of Preventive Veterinary Medicine, College of Veterinary Medicine, Northwest A&F University, Yangling, 712100 Shaanxi China; 2 Scientific Observing and Experimental Station of Veterinary Pharmacology and Diagnostic Technology, Ministry of Agriculture, Shaanxi, 712100 China

**Keywords:** Fenobody, Ranbody, Reporter-nanobody fusions, Sandwich enzyme-linked immunosorbent assay, NDV

## Abstract

**Background:**

Traditional sandwich enzyme-linked immunosorbent assay (ELISA) using polyclonal and monoclonal antibodies as reagents presents several drawbacks, including limited amounts, difficulty in permanent storage, and required use of a secondary antibody. Nanobodies can be easily expressed with different systems and fused with several tags in their tertiary structure by recombinant technology, thus offering an effective detection method for diagnostic purposes. Recently, the fenobody (ferritin-fused nanobody) and RANbody (nanobody-fused reporter) have been designed and derived from the nanobody for developing the diagnostic immunoassays. However, there was no report about developing the sandwich ELISA using the fenobody and RANbody as pairing reagents.

**Results:**

A platform for developing a sandwich ELISA utilizing fenobody as the capture antibody and RANbody as the detection antibody was firstly designed in the study. Newcastle disease virus (NDV) was selected as the antigen, from which 13 NDV-specific nanobodies were screened from an immunized Bactrian camel. Then, 5 nanobodies were selected to produce fenobodies and RANbodies. The best pairing of fenobodies (NDV-fenobody-4, 800 ng/well) and RANbodies (NDV-RANbody-49, 1:10) was determined to develop the sandwich ELISA for detecting NDV. The detection limits of the assay were determined to be 2^2^ of hemagglutination (HA) titers and 10 ng of purified NDV particles. Compared with two commercial assays, the developed assay shows higher sensitivity and specificity. Meanwhile, it exhibits 98.7% agreement with the HA test and can detect the reference NDV strains belonging to Class II but not Class I.

**Conclusions:**

In the presented study, the 13 anti-NDV nanobodies binding the NDV particles were first produced. Then, for the first time, the sandwich ELISA to detect the NDV in the different samples has been developed using the fenobody and RANbody as reagents derived from the nanobodies. Considering the rapidly increasing generation of nanobodies, the platform can reduce the cost of production for the sandwich ELISA and be universally used to develop assays for detecting other antigens.

## Background

The double-antibody sandwich enzyme-linked immunosorbent assay (ELISA) is preferentially used to detect pathogenic bacteria [1], viruses [2], and biomarkers in samples for rapid and accurate diagnosis [3]. For example, many commercial double-antibody sandwich ELISAs have been developed for the diagnosis of human and animal diseases [4, 5]. To develop this assay, the use of capture and reporter-labeled detection antigen-specific antibodies is essential and must be produced in the initial step [6, 7]. While most sandwich ELISA kits and housed-methods employ conventional polyclonal and monoclonal antibodies as indispensable reagents, they present several drawbacks, including limited amounts, difficulty in permanent storage, and required use of a secondary antibody [8, 9]. Hence, there is an urgent need to develop strategies for producing smaller size recombinant antibodies that are more easily produced, selected, and manipulated.

Unlike conventional antibodies, nanobodies are derived from the *Camelidae* heavy chain-only antibodies (VHH) [10], which possess a unique structure and characteristics, including small size (~ 15 kDa), good stability and solubility, high specificity and flexibility [11, 12]. They can be screened from the VHH libraries through phage display technology and panning methodologies and produced by the different expression system [13, 14]. Also considering that they are usually genetically modified by conjugating with reporters at a relatively low cost, traditional antibody-based immunoassays face certain challenges that can be overcome by nanobodies [12, 15]. For instance, the coding sequences of nanobodies are short (approximately 330 bp) and can be directly saved in the computer for a long time, then can be simply re-synthesized for expression before the next use. Based on these advantageous features, nanobodies have been increasingly exploited in the development of biological diagnostics and therapies [12, 16]. However, there are few reports on the development of sandwich ELISA using genetically modified nanobodies as a diagnostic tool [11, 17].

**Scheme 1:**
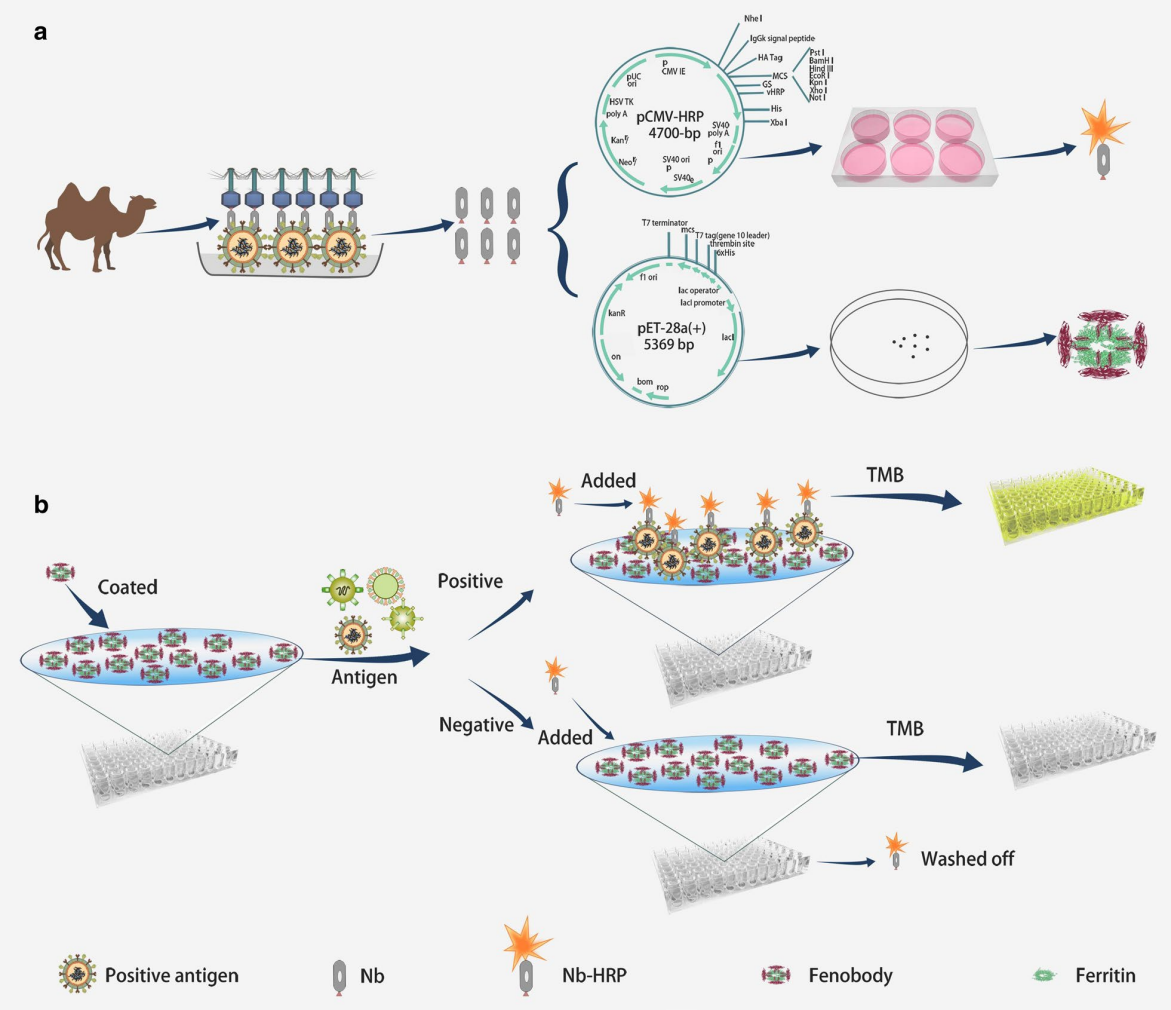
Schematic representation of developing the sandwich ELISA to detect antigens using the fenobody and RANbody as reagents. **a** The platform for expressing fenobody and RANbody. **b** Development of the sandwich ELISA to detect antigen using the fenobody and RANbody as reagents

In the present study, the Newcastle disease virus (NDV) was selected as the antigen to design the sandwich ELISA using the nanobody as the reagents. NDV is one of the most severe pathogenic diseases that has detrimentally affected poultry worldwide [18]. Currently, the hemagglutination (HA) test, hemagglutination inhibition (HI) test, and molecular identification after virus isolation are considered the gold standard methods for the diagnosis of NDV infection [19, 20]. However, these methods are usually time-consuming and require a cumbersome operation [21]. In recent years, many commercial ELISA kits based on viral antigens and NDV-specific conventional antibodies have also been applied for the rapid diagnosis of NDV [22]. However, there are still limited to detect NDV particles from the tissues using the commercial ELISA kit because of their sensitivity [23]. Nanobody as the reagents for developing the sandwich ELISA can overcome the drawback of the commericial ELISA kit. In a previous study, the fenobody (ferritin-fused nanobodies) can apparently enhance the affinity of univalent nanobody against H5N1 avian influenza virus (AIV) [24]. Then, the sandwich ELISA for detecting H5N1 virus using the fenobody as capture antibody can be significantly improved the sensitivity. Yet, procedures of preparation, purification and reporter labelling of traditional antibodies are complicated and costly, which is a heavy burden for the development of commercial ELISA kits [9, 25]. The RANbodies (nanobodies-fused reporter protein) [26, 27] derived from nanobody can also overcome the drawback and there are no need to be reporter labelled the traditional antibodies. But to date, there are few reports about developing sandwich ELISA to detect NDV using nanobody as reagents [28]. Then, in this study, NDV specific nanobodies were obtained from a Bactrian camel immunized with inactivated NDV vaccines. And, fenobodies and RANbodies against NDV were produced through genetic modification of the anti-NDV nanobodies (Scheme 1a). For the first time, a fenobody was implemented as the capture antibody and RANbody as the detection antibody to develop a sensitive, specific, and easy production sandwich ELISA for detection of NDV in the samples (Scheme 1b). We believe that the developing sandwich ELISA based on the fenobodies and RANbodies can be widely used for the analytical detection of many other antigens. Meanwhile, the developed assay displays great developmental prospect for further commercial production and application.

## Materials and methods

### Cells, virus and vectors

HEK293T cells were purchased from the American Type Culture Collection (ATCC) (Manassas, VA, USA) and cultured in Dulbecco’s Modified Eagle’s Medium (Life Technologies Corp, USA) supplemented with 10% fetal bovine serum (FBS, Gibco USA) and penicillin/streptomycin at 37 °C in 5% CO_2_. The NDV strain (LaSota) viral stock was propagated in 9- to 11-day-old SPF chicken embryos [29]. This representative strain belonging to Class II was attenuated one. The allantoic fluid with high HA titers was clarified by centrifugation at 6000*g* for 20 min at 4 °C, applied to 10–50% sucrose gradient, then centrifuged at 110,000*g* for 5 h for purification at 4 °C as described in a previous study [30]. The purified NDV particles (1 mg mL^−1^) were suspended in phosphate buffer saline (PBS, 0.01 mol L^−1^, pH 7.2) and were used as the coating antigen to screen the nanobodies. In addition, the NDV Class II virulent reference strain F48E9 (Genotype IX, GenBank accession number: MG456905, 15,192 bp in length), Class II virulent clinical strain sx10 (GenBank accession number: KC853020, 15,192 bp in length), and Class I reference strain QH-1 (GenBank accession number: KT223818, 15,198 bp in length) were used to determine the ability of the developed assay for detecting different NDV strains. To construct the VHH library, the pMECS vector was kindly provided by Professor Muyldermans and used as described in the previous procedures [13]. To express the nanobody and produce fenobody in the bacterial system, the expression vector pET-28a (Novagen, USA) was used. To produce RANbody, the pCMV-N1-HRP vector was constructed using the pCMV-N1-EGFP (Clontech, Japan) as a backbone based on previous descriptions [26].

### Bactrian camel immunisation and library construction.

A healthy 4-year-old male Bactrian camel was immunized through the subcutaneous route with the commercial inactivated vaccine containing the inactive NDV (LaSota) strain (PULIKE biological engineering company, Luoyang, China). The Bactrian camel was injected a total of four times, which was performed at two-week intervals. The titration of the antibody against NDV in the serum samples was detected with an indirect ELISA based on a previous description [26, 31, 32], except the purified NDV particles (200 ng/well) were employed as the coating antigen. Five days after the last immunisation, the lymphocytes were isolated from the collected blood by Leucosep^®^ tubes (Greiner Bio-One, Germany). Then, total cellular RNA was extracted, and the cDNA was synthesized using the oligo-dT_12–18_ primer with the SuperScript™ II Reverse Transcriptase based on the instructions. The VHH sequences from the cDNA pool were amplified in two rounds using nested PCR (Additional file 1: Table S1), and a VHH phage display library was constructed based on the procedures as previously described [31]. After identification using the bacterial PCR with the primer pairs MP57 and GIII (Additional file 1: Table S1), the library was stored at − 80 °C in LB medium supplemented with 20% w/v glycerol until used.

### Screening of specific nanobodies against NDV

Phage rescue and biopanning were performed as described previously [13]. In biopanning, 1 µg purified NDV particles (LaSota strain) were used as the coating antigen. After three rounds of biopanning, the enrichment of specific phage particles was evaluated with polyclonal phage ELISA. Then, 96 colonies were randomly picked, induced with isopropyl-β-d-thiogalactoside (IPTG, 1 mM), and their periplasmic extracts were tested by indirect ELISA for the presence of NDV-specific nanobodies. All positive clones contained VHH genes were sequenced and grouped according to their complementary determining regions 3 (CDR3) sequence. In addition, the binding activities of different nanobodies were evaluated with indirect ELISA using different dilutions of periplasmic extracts as the first antibody.

### Selection of the NDV-specific nanobodies for producing fenobodies and RANbodies

To select the nanobodies for subsequently producing fenobodies and RANbodies, a capture ELISA was designed. Briefly, the ELISA plate was coated with the different periplasmic extracts containing the NDV-specific nanobodies and incubated 12–14 h at 4 °C. Each nanobody was coated into the two wells. Purified NDV particles (1 µg/well, positive control) and PBS (negative control) were separately added to the two wells of each nanobody after they were blocked and washed. Then, the monoclonal antibody against NDV (QIANXUN Biotech Company, Guangzhou, China) was added in 1:1000 dilutions and incubated for 1 h at 37 °C. After washed again, the HRP-goat anti-mouse IgG antibody (TransGen Biotech Company, Beijing, China) was added, and the reaction was colored with tetramethylbenzidine (TMB) [A: 205 mmol L^−1^ potassium cirate (pH 4.0); B: 41 mmol L^−1^ tetramethyl benzidine; A:B (v/v) = 39:1]. After the reaction was stopped using 3 mol L^−1^ H_2_SO_4_, the optical density at 450 nm (OD_450nm_ value) was read using an automatic ELISA plate reader. Then, the coated nanobodies emerging from the positive OD_450nm_ value/negative OD_450nm_ value (P/N) > 3.0 were selected for producing fenobodies and RANbodies.

### Preparation of fenobodies against NDV

The fenobodies were expressed according to the previous descriptions with some modifications [24]. The complete gene encoding ferritin based on the sequences of *P. furiosus* was provided by GENWIZ Biotech Company. According to the above obtained sequences encoding the nanobody against NDV and ferritin, the two primer pairs (Additional file 1: Table S1) were designed. One pair was used to amplify the selected VHH genes, and the other was utilized to amplify the truncated gene encoding from the 1 to 146 amino acid (aa) region of ferritin. Meanwhile, some overlapping gene sequences were designed in the reverse and forward primers to amplify the nanobodies and ferritin, respectively (Additional file 1: Table S1). Then, overlapping PCR was used to amplify the fusion gene, in which nanobodies replaced helix ε and loop (147–174 aa) of ferritin by GS linker (GGGS)_3_. Subsequently, to construct the recombinant plasmid (pET-28a-fenobody), the fusion genes were digested with the enzymes NdeI and BamHI (TaKaRa, Japan) and then were ligated into the pET-28a vector digested with the same two enzymes. After identification, the fenobody was expressed in the transformed Transetta (DE3) *E. coli* (TransGen Biotech, China) by adding 0.1 mmol L^−1^ IPTG at 16 °C. Then, the pellets were resuspended in lysis buffer (NaH_2_PO_4_·2H_2_O 7.80 g, 50 mmol L^−1^, NaCl 29.22 g, 500 mmol L^−1^, imidazole 0.68 g, 10 mmol L^−1^, pH 8.0). After sonication on ice, the soluble fenobody with His tags was loaded onto Ni–NTA-6FF Column (Smart-Lifesciences, Changzhou, China) and Capto Core 700 chromatography (GE Healthcare BioSciences AB, Uppsala, Sweden) for purification. Based on the manual instructions of Capto Core 700 chromatography, only the large biomolecules can directly flow and the small ones enter into the core. In addition, an anti-H9N2 nanobody was designed to produce fenobody as the negative control. After purification, SDS-PAGE and Western blot assays were employed to analyze the expression and purification of the fenobody, while the purified fenobodies were frozen at − 20 °C in 1 mmol PMSF and 0.02% w/v NaN_3_. In addition, the fenobodies were negatively dyed with 2% uranium acetate and observed at TEM (JEM-1400) whether fenobody self-assembled into 24 subunit nanocage. To verify the fenobody binding to the NDV particles, indirect ELISA was used with the purified NDV particles as the coated antigen. The procedure for the indirect ELISA is characterized below.

### Affinity and half-life extension test of fenobody

To compare the affinity with the NDV particles and half-life extension of the fenobody with univalent or traditional nanobody, traditional nanobodies were expressed and purified based on a previous description [31]. Briefly, the VHH genes encoding the different nanobodies were directly ligated into the pET-28a vector. The nanobodies were expressed in *E.coli* BL21 (DE3) and purified with the Ni–NTA resin according to the manufacturer’s instruction. Then, the purified traditional nanobodies were analyzed with SDS-PAGE.

To compare the affinity of the fenobody and traditional nanobody, a capture ELISA were designed and performed based on a previous characterisation with some modifications [24, 33, 34]. Briefly, after the same amounts of fenobodies and traditional nanobodies (800 ng/well) were coated into the ELISA plates, NDV particles (1 µg/well), anti-NDV monoclonal antibodies (1 mg mL^−1^, 1:2000, 100 µL/well), and HRP labeled goat anti-mouse antibodies (1 mg mL^−1^, 1:5000, 100 µL/well) were then added to the plates one by one. Then the molar ratio of the four reagents added in the ELISA plate was 180:1.5:2.5:1. To accurately calculate the parameters of the binding reaction using GraphPad Prism 5 software, the above capture ELISA first employed the fenobody (used in the developed sandwich ELISA) and the corresponding nanobody as the coating antigens with the amount of 800 ng/well. Then, the same reagents were added into the wells, except that 100 µL allantoic fluid containing NDV with HA titers (2^12^ to 2^0^) was used instead of NDV particles (1 µg/well).

To analyze the half-life extension, the fenobody and traditional nanobody were both labeled with FITC based on the descriptions by Fan K et al. [24] and Fisher et al. [35]. In brief, the 200 nM FITC (Sigma-Aldrich, USA) was labelled to 50 nM fenobody/nanobody in 1 mL of carbonate/bicarbonate buffer (100 mM carbonate, pH 9.0). The FITC-labeled fenobody and FITC-labeled traditional nanobody were intravenously injected into female BALB/c mice (FITC 2 nmols/mouse) via the tail vein. Then, the blood was collected from the tail vein at different time points, including 20, 30, 40, 60, 120, 240, 360, 720, 1200, and 1440 min. The fluorescence of the FITC-protein in the blood was determined by VICTOR^™^ X Series Multilabel Plate Readers (PerkinElmer, USA) with excitation wavelength setting at 485 nm and emission wavelength at 535 nm (Ex485/Em535), 1.0 s [36]. Then, the values were analyzed using the Origin software.

### Preparation of RANbodies against NDV

To produce RANbodies against NDV, the method described by Sheng et al. was followed using HRP as the reporter [26]. Briefly, the modified vector pCMV-N1-HRP and positive phagemid pMECS containing the genes encoding nanobodies were digested with both PstI and NotI enzymes. Then, the nanobody genes were ligated into the vector pCMV-N1-HRP using a DNA Ligation Kit according the manufacturer’s instructions (TaKaRa, Japan). The positive recombinant plasmids were confirmed by sequencing and used for transfection. Then, the HEK293T cells were transfected with the positive plasmids. After 72 h of transfection, the medium containing the secreted RANbodies was harvested, and supplemented with 0.02% w/v NaN_3_ for direct use. In addition, to avoid the exogenous pollution, a nanobody against H9N2 was also selected to produce RANbody as the negative control.

The expressions of RANbodies in the HEK293T cells and in the medium were determined using indirect immunofluorescence assay (IFA) and Western blot assay, respectively. The two assays both employed anti-His monoclonal antibody as the first antibody. The FITC and HRP-labeled goat anti-mouse antibodies were separately used as the second antibodies for the IFA and Western blot assay, respectively. In addition, the specific binding with NDV and titers of RANbodies in the medium were identified by direct ELISA using purified NDV particles as the coated antigen.

### Indirect ELISA

Indirect ELISA was used to analyze the specific binding of periplasmic extracts from 96 clonal *E.coli*, fenobodies, and RANbodies with NDV. Briefly, the 96-well Maxisorp microtiter plates (Nucn-Immunoplate, Roskilde, Denmark) were coated with the purified NDV particles (LaSota strain, 200 ng/well) and incubated at 4 °C overnight. The purified H9N2 AIV particles (200 ng/well) were used as the negative control. After three more washings with PBS containing 0.5% w/v Tween-20 (PBS’T), the periplasmic extracts, fenobodies, and RANbodies were separately added into the wells and incubated for 1 h at RT. After washing three times, anti-His tag monoclonal antibodies (1 mg mL^−1^, 1:2000, 100 µL/well) and HRP-goat anti-mice IgG antibodies (1 mg mL^−1^, 1:5000, 100 µL/well) were subsequently added into the wells containing periplasmic extracts and fenobodies. After another three washes, TMB (100 μL/well) was added and incubated in the dark for 15 min at RT. For the RANbodies (nanobodies fusion with HRP), the TMB was directly added into the wells without the first and second antibodies to initiate a color reaction. The color reaction was stopped with 3 mol L^−1^ H_2_SO_4_ (50 μL/well), and the OD_450nm_ values were read using an automatic ELISA plate reader (BIO-RAD).

### Development of the fenobody and RANbody-based sandwich ELISA

To develop the sandwich ELISA, the fenobodies and RANbodies were separately used as the capture and detection reagents, respectively. To obtain the best pair, the ELISA plate was coated with the 800 ng/well of different fenobodies and incubated at 4 °C overnight. After the plate was washed three times with PBS’T, the purified NDV particles (LaSota strain, 1 µg/well), as the positive (P) control, and H9N2 AIV, as the negative (N) control, were separately added for 1 h after blocking with 5% w/v skim milk in PBS’T for 1 h. After washed again, 100 µL of different RANbodies was added into the well and incubated for 1 h at RT. After a final washing, TMB was added to produce the color reaction, and the OD_450nm_ values were read after the reaction was stopped with 3 mol L^−1^ H_2_SO_4_. The best pair of nanobodies was selected at the highest P/N value.

Secondly, the optimal amounts of capture fenobody and detecting RANbody for the sandwich ELISA were determined using a checkerboard titration based on previous studies [26, 34]. Different amounts of the fenobody (100, 200, 400, 800 and 1000 ng/well) and different dilution ratios of RANbodies (1:1, 1:10, 1:100, 1:1000, and 1:10,000) were used in the sandwich ELISA. Same amounts of purified NDV particles (1 µg/well) and H9N2 AIV were used. Then, the optimal amounts of fenobody and RANbody were determined when the P/N value was the highest.

Further, the incubation times between fenobody and NDV and between NDV and RANbody were optimized. The incubation time of fenobody capturing NDV was tested for 20, 40, 60, 80, and 100 min, while the incubation time of RANbody and NDV was examined for 30, 60, and 90 min. Using H9N2 AIV as the negative control and checkerboard titration, when the P/N ratio was highest, the two optimal times were determined.

The procedures of the developed sandwich ELISA were performed as follows. First, the ELISA plate was coated with the NDV-fenobody-4 using the above optimized amount in PBS at 4 °C overnight. On the second day, the plate was blocked with the 2.5% skim milk (200 µL/well) and incubated at RT for 1 h. After washed three times with PBS’T (300 µL/well), different testing samples (100 µL/well) were added to the wells in the plate, which were then incubated at RT for optimized times. After washing three more times, NDV-RANbody-49 with the optimized dilution was added to the plate then incubated at RT for optimized times. After the plate was washed a final three times, TMB (100 µL/well) was used to induce the color reaction. Finally, the plate was read at OD_450nm_ with an automatic ELISA plate reader after the addition of 3 mol L^−1^ H_2_SO_4_ (50 μL/well).

### Validation of the developed sandwich ELISA

To determine the cut-off value of the sandwich ELISA, 150 negative samples, including tracheal and cloacal swabs (n = 45), allantoic fluid from SPF chicken embryo (n = 25), cell culture medium (n = 25), chicken sera (n = 25) and tissue samples from SPF chickens including liver, lung, kidney, spleen, thymus and trachea (n = 30), were tested. Generally, NDV is detected from these above samples in clinical trials. The tracheal and cloacal swabs were washed with 20–50 µL PBS, then tissue samples were grinded, freeze-thawed three times, and centrifuged using the supernatant. The cut-off value was the mean of the OD_450nm_ values of 150 negative samples + 3 times standard deviations (SD) tested using the developed sandwich ELISA [26, 34]. Different amounts (0 to 1000 ng) of the purified NDV particles and different HA titers (2^8^ to 2^0^) of allantoic fluid containing NDV were both detected with the sandwich ELISA to determine the limited viral amount of the assay. Based on the OD_450nm_ values of sandwich ELISA and HA titres of allantoic fluid, the correlation between sandwich ELISA and HA test was calculated using Microsoft Excel. The correlation between the sandwich ELISA and amounts of purified NDV particles was also calculated based on the OD_450nm_ values and different amounts of NDV.

To determine the specificity of the sandwich ELISA, other poultry disease viruses, including H9N2 AIV, H5N1 AIV, H7N9 AIV, infectious bronchitis virus (IBV), infectious bursal disease virus (IBDV), fowl adenovirus (FADV), and J subgroups of avian leukosis virus (ALV-J) were also detected using the assay.

In addition, to determine if the developed sandwich ELISA can be used to detect different NDV strains, reference strains F48E9 (Class II virulent strain), QH-1 (Class I strain), and clinical isolate sx10 (Class II virulent strain) were selected. Meanwhile, to evaluate whether the dead NDV was detected by the developed sandwich ELISA or not, the different NDV strains were inactivated with 5% formaldehyde at 4 °C for 7 days [37]. Then, the inactivated NDV strains were detected with the developed sandwich ELISA.

### Application of the developed sandwich ELISA for detecting NDV in chicken tissue samples

To verify that the developed sandwich ELISA can be used to detect NDV from different tissue samples, an animal experiment was designed, and different tissue samples were collected from the chickens infected with NDV.

Twenty 30-day-old specific-pathogen-free (SPF) chickens were randomly divided into two groups. One group (n = 10) was infected with NDV strain F48E9 stock containing 2^5^ HA titers using the nasal route. The second group was inoculated with PBS as the negative control. After challenged, two chickens each were necropsied at 3, 4, 5, 7, and 10 days post inoculation (dpi). A total of 340 swab samples (170 for each group) were collected from the thymus, pancreas, proventriculus, liver, spleen, kidney, small intestine, large intestine, cecal tonsil, feces, brain, trachea, lung, throat swab, tracheal swab, bursa, and cloacal tissues. Then, the same amounts of collected tissue samples were grinded. After freeze-thawing three times, 100 µL suspensions of each tissue sample were used to detect NDV using the developed sandwich ELISA.

### Comparisons of the developed sandwich ELISA with other commercial methods

As described above, all 340 tissue samples from the animal experiment and four different NDV strains (LaSota, F48E9, sx10, and QH-1) were further used to detect NDV with a commercial monoclonal antibody-based sandwich ELISA kit (Yoyong Biotechnology Company, Guangzhou, China) and a commercial immune colloidal gold strip (Yoyong Biotechnology Company, Guangzhou, China). The coincidence rates of the developed sandwich ELISA with the monoclonal antibody-based sandwich ELISA kit and immune colloidal gold strip were calculated using Microsoft Excel’s CORREL function. In addition, the 367 samples from the clinical chickens, including 189 tracheal and cloacal swabs and 178 tissue samples, were also tested with the three assays, and the coincidence rates were calculated.

In addition, the above positive samples detected by the developed sandwich ELISA were used to inoculate the SPF chicken embryos by allantoic cavity route. Then, the allantoic fluid samples were collected and detected using the HA test. The coincidence rates of the two assays were also calculated using the Microsoft Excel’s CORREL function.

### Statistical analysis

All experiments were repeated at least three times. Kappa index values were calculated to estimate the coincidence between the developed sandwich ELISA and the monoclonal antibody-based commercial ELISA kit, commercial immune colloidal gold strip, and HA test. These calculations were performed using SPSS software (Version 20, https://www.spss.com.cn).

## Results

### Screening and sequencing of nanobodies against NDV

**Fig. 1:**
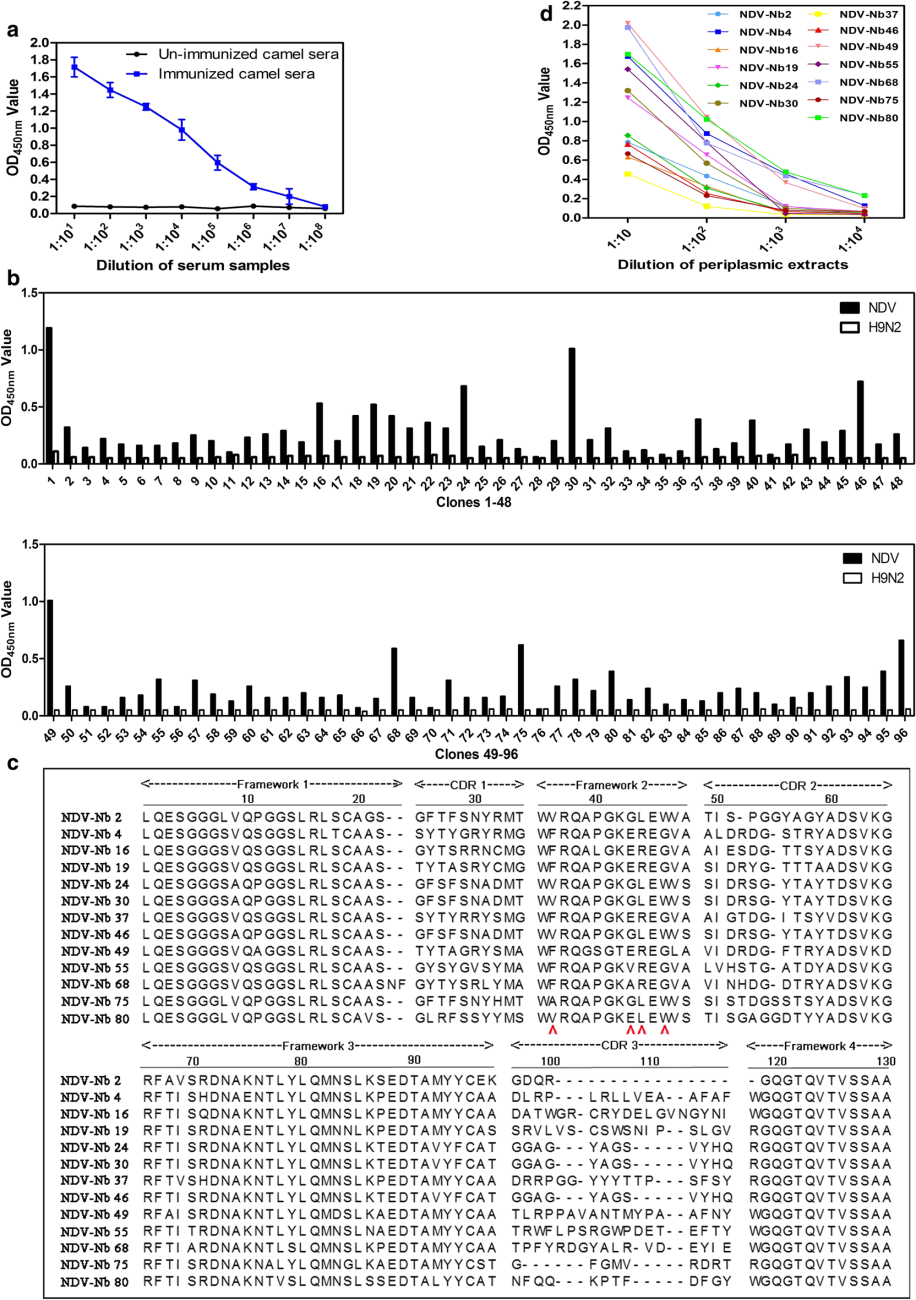
Screening the NDV-specific nanobodies from the VHH library. **a** Titers of antibodies against NDV particles in the sera from the camel after the fifth immunisation. **b** Detection of the periplasmic extracts from 96 clones reacting with NDV particles with indirect ELISA. **c** Alignment of amino acid sequence of 13 screened nanobodies. Numbering and CDRs according to previous methods. The residues at positions 37, 44, 45, and 47 are indicated by red arrows. **d** Titration of the 13 screened nanobodies in the periplasmic extracts binding with NDV

After the last immunisation, the titres of anti-NDV antibodies in the immunized camel reached 1:10^6^ detecting by the indirect ELISA (Fig. 1a). Then, the lymphocytes were isolated from the camel to extract total cellular RNA and a target band of about 400 bp in size was amplified using reverse transcription nested PCR using the extracted RNA as a template (data not shown). Finally, a phage display VHH library against NDV particles, containing approximately 3 × 10^8^ individual transformants, was constructed. More than 98% of these colonies had insertion fragments corresponding to a VHH gene (approximately 400 bp), as determined by colony PCR (Additional file 1: Fig. S1). Sixty randomly selected clones were sequenced, with each clone containing a distinct VHH sequence (data not shown).

After three rounds of phage particle biopanning using purified NDV particles as coating antigen with the indirect ELISA, the phage particles binding NDV was enriched (Table 1). Then, the 96 individual colonies containing the VHH genes from the third round biopanning plate were selected and expressed induced for VHH expression by IPTG. The periplasmic extracts from the 96 colonies were extracted and identified whether binding to purified NDV particles in an indirect ELISA. Out of the 96 extracts, the 70 ones were specifically bound with the NDV particles (Fig. 1b). After they were sequenced by the company for the VHH genes, sequences analysis based on CDR3 regions of VHH genes revealed that 13 unique nanobodies were screened (Fig. 1c). Among which, NDV-Nb4, -Nb19, -Nb30, -Nb49, -Nb55, -Nb68, and -Nb80 showed the higher binding activity (Fig. 1d).

**Table 1:** Enrichment of nanobodies against NDV particles from the phages during three rounds of panning

Round of panning	Phage input (PFU/Well)	Phage output (PFU/Well)	N output (PFU/Well)	Enrichment
1st round	5 × 10^10^	6.3 × 10^5^	3.5 × 10^4^	1.8 × 10^1^
2nd round	5 × 10^10^	2.4 × 10^8^	6 × 10^4^	4 × 10^3^
3rd round	5 × 10^10^	3.59 × 10^7^	1 × 10^3^	3.59 × 10^4^

### Selection of nanobodies for expression of fenobody and RANbody

From the 13 nanobodies chosen to produce fenobodies and RANbodies, the results of capture ELISA showed that the P/N values were above 3 when the periplasmic extracts from 2, 4, 24, 30, and 49 clones were used as the coating antigen in the capture ELISA (Table 2). Then, these clones were selected as templates for subsequently producing fenobodies and RANbodies.

**Table 2:** Analysis of the 13 nanobodies against NDV capturing viral particles using capture ELISA

Samples	Periplasmic extracts from different clones as coated antigen for the capture ELISA												
	2	4	16	19	24	30	37	46	49	55	68	75	80
P	0.467	0.576	0.238	0.356	0.654	0.378	0.287	0.156	0.601	0.189	0.201	0.367	0.265
N	0.098	0.076	0.094	0.132	0.101	0.097	0.098	0.065	0.067	0.076	0.095	0.133	0.092
P/N	4.77	7.58	2.53	2.70	6.48	3.90	2.93	2.4	8.97	2.49	2.12	2.76	2.88

The same amounts of periplasmic extracts from 13 clones were used as the capture antibody. NDV particles were used as the positive control (P) and H9N2 AIV as the negative control (N)

### Expression and purification of fenobodies against NDV

**Fig. 2:**
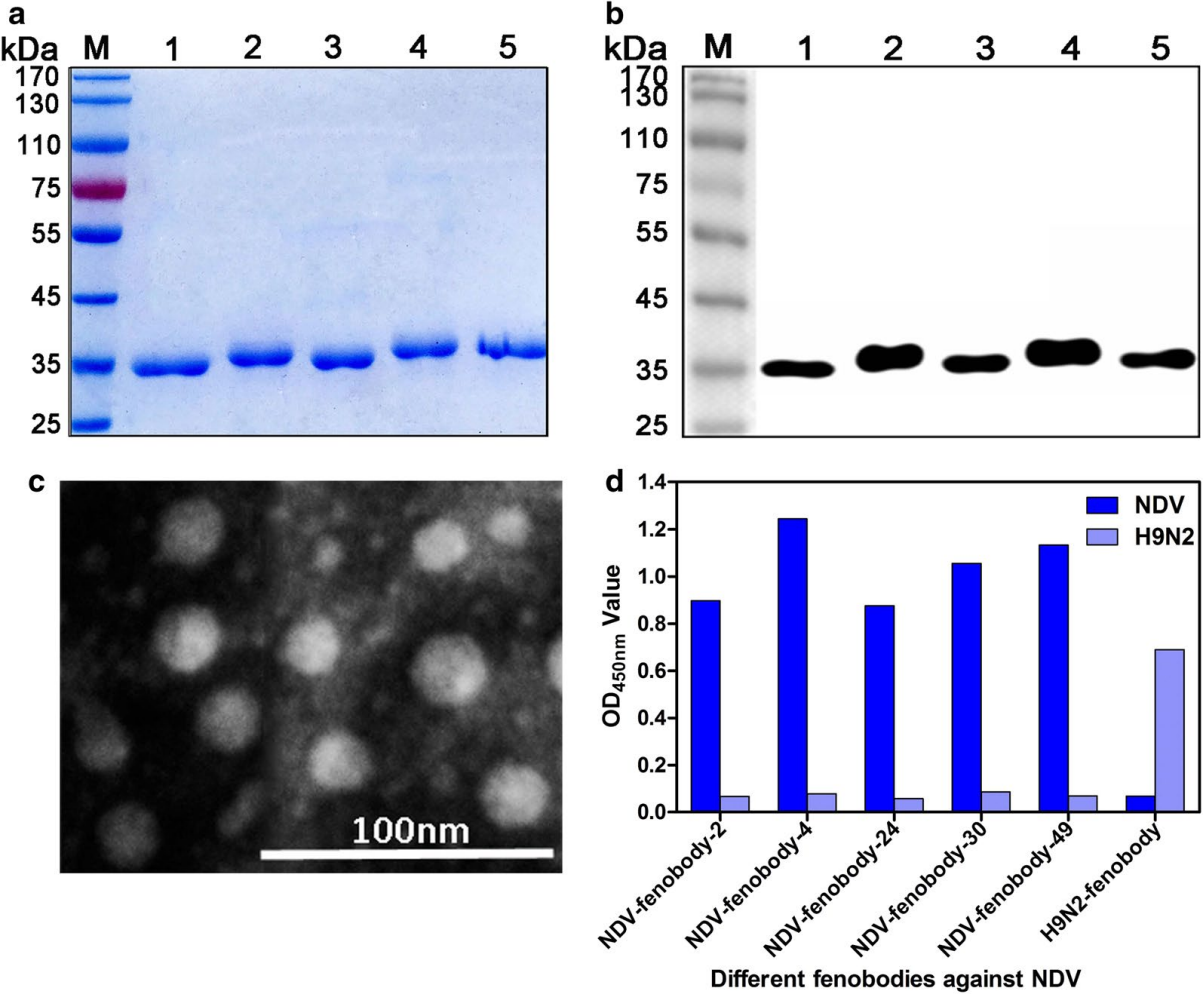
Production and characterization of the 5 fenobodies against NDV particles. **a** SDS-PAGE analysis of the 5 fenobodies against NDV with bacterial system for expression and with Ni–NTA-6FF Column for purification. Lanes 1–5: NDV-fenobody-2, -4, -24, -30 and -49. **b** Western blot analysis of the purified 5 fenobodies against NDV reacting with anti-His monoclonal antibody. Lanes 1–5: NDV-fenobody-2, -4, -24, -30 and -49. **c** TEM analysis of the 5 purified fenobodies self-assembled into the 24 subunit nanocage. A picture of the NDV-fenobody-4 is shown here, which is same as 4 other fenobodies. **d** Analysis of the 5 fenobodies specifically binding with the NDV particles with indirect ELISA and the anti-H9N2 fenobody as the negative control

The 2, 4, 24 30, and 49 clones were used as templates to produce fenobodies. After expressed with the bacterial system, the five fenobodies were expressed as soluble proteins with a size of approximately 34 kDa as expected, which were predicted by the EditSeq program of Lasergene 7.1 software based on the amino acids of fenobodies. After purification with Ni–NTA columns, SDS-PAGE and Western blot analysis showed that 5 high purity fenobodies were obtained and can react with the anti-His monoclonal antibody (Fig. 2a, b). These fenobodies were respectively named NDV-fenobody-2, -4, -24 -30, and -49. Then, the fenobodies were purified with Capto Core 700 chromatography and analyzed by negative staining TEM, which revealed that they resemble the cage-like architecture of ferritin (Additional file 1: Fig. S2 and Fig. 2c). The results of indirect ELISA showed that the 5 purified fenobodies can specifically bind with the purified NDV particles and the anti-H9N2 fenobody cannot bind (Fig. 2d).

### Fenobodies exhibit high affinity to NDV and half-life extension

**Fig. 3:**
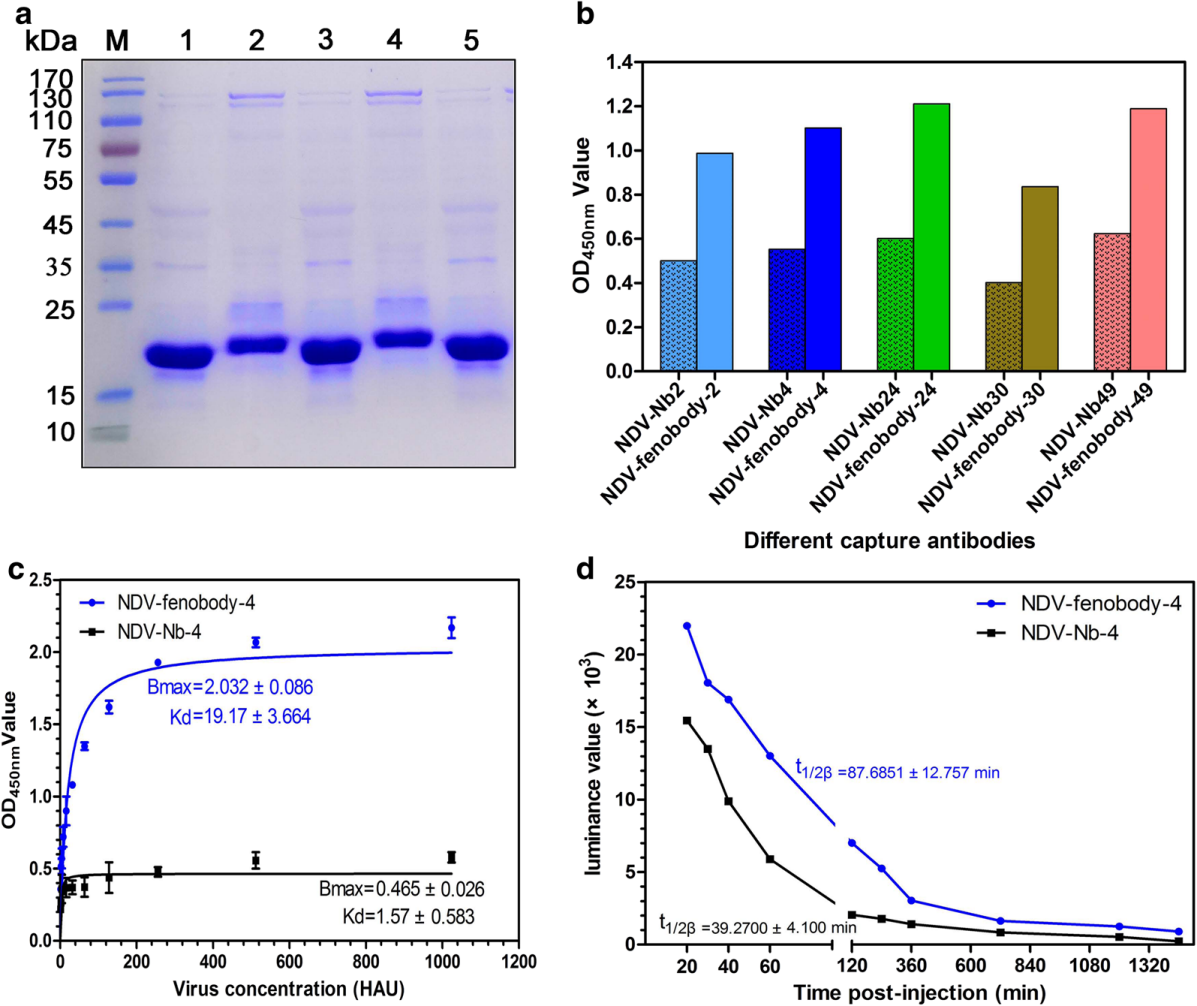
Comparisons of affinity and high-life extension between fenobody and traditional nanobody. **a** SDS-PAGE analysis of the 5 traditional nanobodies against NDV expressed by bacterial system and purified with Ni–NTA-6FF Column. Lanes 1–5: NDV-Nb-2, -4, -24, -30 and -49. **b** Comparisons of the 5 fenobodies and traditional nanobodies binding the NDV with a capture ELISA. **c** Binding affinity analysis of NDV-fenobody-4 and NDV-Nb-4 to NDV particles by capture ELISA. **d** Concentrations of NDV-fenobody-4 and NDV-Nb-4 in the blood from the different time points of the injected BABL/c

The 5 traditional nanobodies were also expressed with the bacterial system and purified with Ni–NTA columns for comparing the binding ability between the fenobody and traditional nanobody with NDV particles. SDS-PAGE analysis showed that these univalent nanobodies were successfully expressed with the expected size of approximately 17 kDa (Fig. 3a) and were separately named NDV-Nb-2, -4, -24, -30, and -49. Compared with the capture ELISA using the NDV-Nb-2, -4, -24 -30, and -49 as the coating antigens, NDV-fenobody-2, -4, -24 -30, and -49 exhibited a higher binding affinity with NDV particles (Fig. 3b). When using the NDV-fenobody-4 and NDV-Nb-4 as the coating antigen, the results revealed that both the fenobody and traditional nanobody bound to the NDV particles, but the apparent affinity of NDV-fenobody-4 was 12.2 times higher than that of NDV-Nb-4. Then, the affinity constants for NDV-fenbody-4 and NDV-Nb4 were separately determined to be 191.7 ± 3.664 HAU mL^−1^ and 15.7 ± 0.583 HAU mL^−1^ by GraphPad Prism 5 software, indicating that NDV-fenbody-4 had a higher affinity to the NDV virus (Fig. 3c).

To analyze the half-life extension of NDV-fenobody-4 and NDV-Nb-4, the fluorescence values in the blood from the injected BABL/c were determined. The results show that the half-life of fenobody in blood is approximately 2.23 times higher than that of monovalent nanobodies, indicating that NDV-fenobody-4 is more stable (Fig. 3d).

### Expression of RANbodies against NDV

**Fig. 4:**
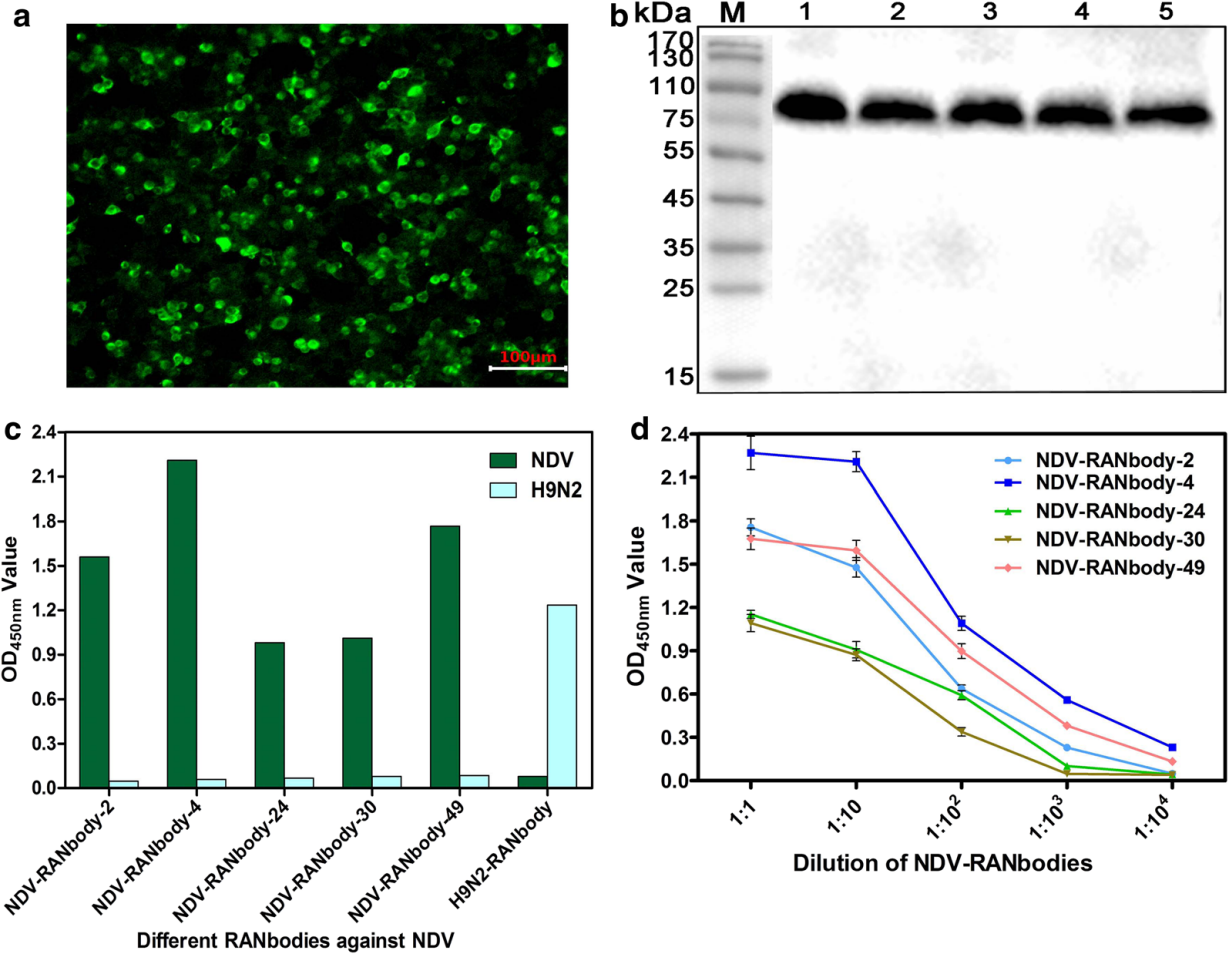
Production and characterisation of the 5 RANbodies against NDV particles. **a** IFA detection of the 5 RANbodies expressed in the HEK293T cells. A picture of the NDV-RANbody-49 is shown here, which is same as other 4 RANbodies against NDV. **b** Western blot analysis of the 5 RANbodies against NDV secreted into the medium of HEK293T cells. The anti-His monoclonal antibody was used as the first antibody and the HRP-goat anti-mouse antibody as the second antibody. Lanes 1–5: NDV-RANbody-2, -4, -24, -30 and -49. **c** Analysis of the 5 RANbodies specifically binding with the NDV particles with direct ELISA and the anti-H9N2 RANbody as the negative control. **d** Titers of the 5 RANbodies in the medium of HEK293T cells using direct ELISA detection

After the 5 recombinant plasmids were transfected into the HEK293T cells, the IFA results showed that the 5 RANbodies were successfully expressed in the HEK293 cells (Fig. 4a). Results of the Western blot analysis revealed that these RANbodies secreted into the medium (Fig. 4b). Moreover, the results of direct ELISA suggest that the 5 RANbodies in the medium can still bind with NDV and the anti-H9N2 RANbody cannot bind (Fig. 4c). The titers of these five RANbodies, named NDV-RANbody-2, -4, -24, -30, and -49, against NDV particles in the medium were determined to be 1:10^3^, 1:10^4^, 1:10^2^, 1:10^2^, and 1:10^4^, respectively (Fig. 4d).

### Development of fenobody and RANbody-based sandwich ELISA for detecting NDV

The same amounts of the 5 fenobodies as the capture antibodies and 5 RANbodies as the detection antibodies were cross-used in the sandwich ELISA. Results show that the P/N value of the developed sandwich ELISA was the highest (39.86) when NDV-fenobody-4 was used as the capture reagent and NDV-RANbody-49 as the detecting reagent (Table 3), suggesting that this specific pair is optimal for the developed sandwich ELISA.

**Table 3:** Selecting the best pairs of fenobody and RANbody for developing the sandwich ELISA

Fenobodies	Samples	RANbodies				
		NDV-RANbody-2	NDV-RANbody-4	NDV-RANbody-24	NDV-RANbody-30	NDV-RANbody-49
NDV-fenobody-2	P	0.060	0.067	1.402	0.065	0.3005
	N	0.062	0.056	0.569	0.055	0.068
	P/N	0.96	1.19	2.46	1.18	4.42
NDV-fenobody-4	P	0.551	0.055	1.011	0.058	*2.272*
	N	0.055	0.058	0.062	0.065	*0.057*
	P/N	10.02	0.95	16.31	0.89	*39.86*
NDV-fenobody-24	P	1.383	0.069	0.067	0.060	0.864
	N	0.064	0.059	0.063	0.059	0.054
	P/N	21.61	1.17	1.06	1.02	16
NDV-fenobody-30	P	1.344	0.073	1.249	0.066	0.534
	N	0.065	0.059	0.056	0.058	0.066
	P/N	20.68	1.24	22.30	2.16	8.09
NDV-fenobody-49	P	0.856	0.069	0.211	0.069	0.058
	N	0.055	0.055	0.06	0.055	0.063
	P/N	15.56	1.25	3.52	1.25	0.92

Different NDV-fenobodies were used as the capture antibody and NDV RANbodies as the detection antibody. NDV particles were as the positive control (P) and H9N2 AIV as the negative control (N). Italic represents the best conditions

The results of checkerboard titration show that the P/N value was highest (44.87) at the optimal conditions of 800 ng/well NDV-fenobody-4, as the capture reagent, and 1:10 dilution of NDV-RANbody-49, as the detection reagent, in the developed sandwich ELISA (Table 4).

**Table 4:** Optimized amount of NDV-fenobody-4 as the capture antibody and dilution of NDV-RANbody-49 in the medium as the detection antibody using the sandwich ELISA

Amounts of NDV-fenobody-4 (ng/well)	Samples	Different dilutions of NDV-RANbody-49				
		1:1	1:10	1:100	1:1000	1:10,000
100	P	1.058	0.945	0.786	0.199	0.058
	N	0.046	0.057	0.061	0.061	0.061
	P/N	23.00	16.58	12.89	3.16	0.95
200	P	1.776	1.782	0.800	0.127	0.056
	N	0.055	0.062	0.065	0.065	0.053
	P/N	32.29	28.74	12.31	2.27	1.06
400	P	2.628	2.315	1.295	0.181	0.06
	N	0.071	0.064	0.055	0.055	0.063
	P/N	37.01	36.17	23.55	3.29	0.95
800	P	2.632	*2.737*	1.417	0.199	0.061
	N	0.065	*0.061*	0.058	0.058	0.073
	P/N	40.49	*44.87*	24.43	3.21	0.84
1000	P	2.360	2.173	1.269	0.161	0.058
	N	0.070	0.051	0.049	0.049	0.066
	P/N	33.71	42.61	25.90	3.04	0.88

NDV was the positive (P) control and H9N2 AIV the negative (N). Italic represents the best conditions

Also using checkerboard titration, the results indicated that the optimized incubation time was 80 min for NDV-fenbody-4 with purified NDV particles and 30 min for NDV-RANbody-49 with NDV particles (Table 5). Then, the procedures of the developed sandwich ELISA were determined and characterized according to the method section using the above optimized conditions.

**Table 5:** Optimized incubation times of NDV-fenobody-4 capturing antigens and the NDV-RANbody-49 detection antigen with the sandwich ELISA

Incubation times of between the testing samples with NDV-RANbody-49 (min)	Samples	Incubation times of between NDV-fenobody-4 with the testing samples (min)				
		20	40	60	80	100
30	P	0.554	0.786	0.588	*1.265*	1.222
	N	0.066	0.070	0.074	*0.068*	0.074
	P/N	8.39	11.23	7.95	*18.60*	16.51
60	P	0.568	0.987	1.063	1.133	1.311
	N	0.077	0.078	0.074	0.087	0.092
	P/N	7.38	12.65	14.36	13.02	14.25
90	P	1.031	1.254	1.169	1.252	1.296
	N	0.078	0.084	0.082	0.076	0.11
	P/N	13.22	14.93	14.26	16.47	11.78

NDV was the positive (P) control and H9N2 AIV the negative (N) control. Italic represents the best conditions

### Cut-off value for the developed sandwich ELISA

The developed sandwich ELISA was utilized to detect NDV in 150 negative samples, including tracheal and cloacal swabs, allantoic fluid, cell culture medium, chicken sera, and tissue samples. The results reveal that the average OD_450nm_ value of the 150 negative samples was 0.0550 with an SD of 0.0115, and the cut-off value of the developed sandwich ELISA was (0.0550 + 3SD). The OD_450nm_ value of the tested samples was above 0.0895 and, thus, considered positive, while conversely, the samples were negative.

### Limitation and specificity of the developed sandwich ELISA

**Fig. 5:**
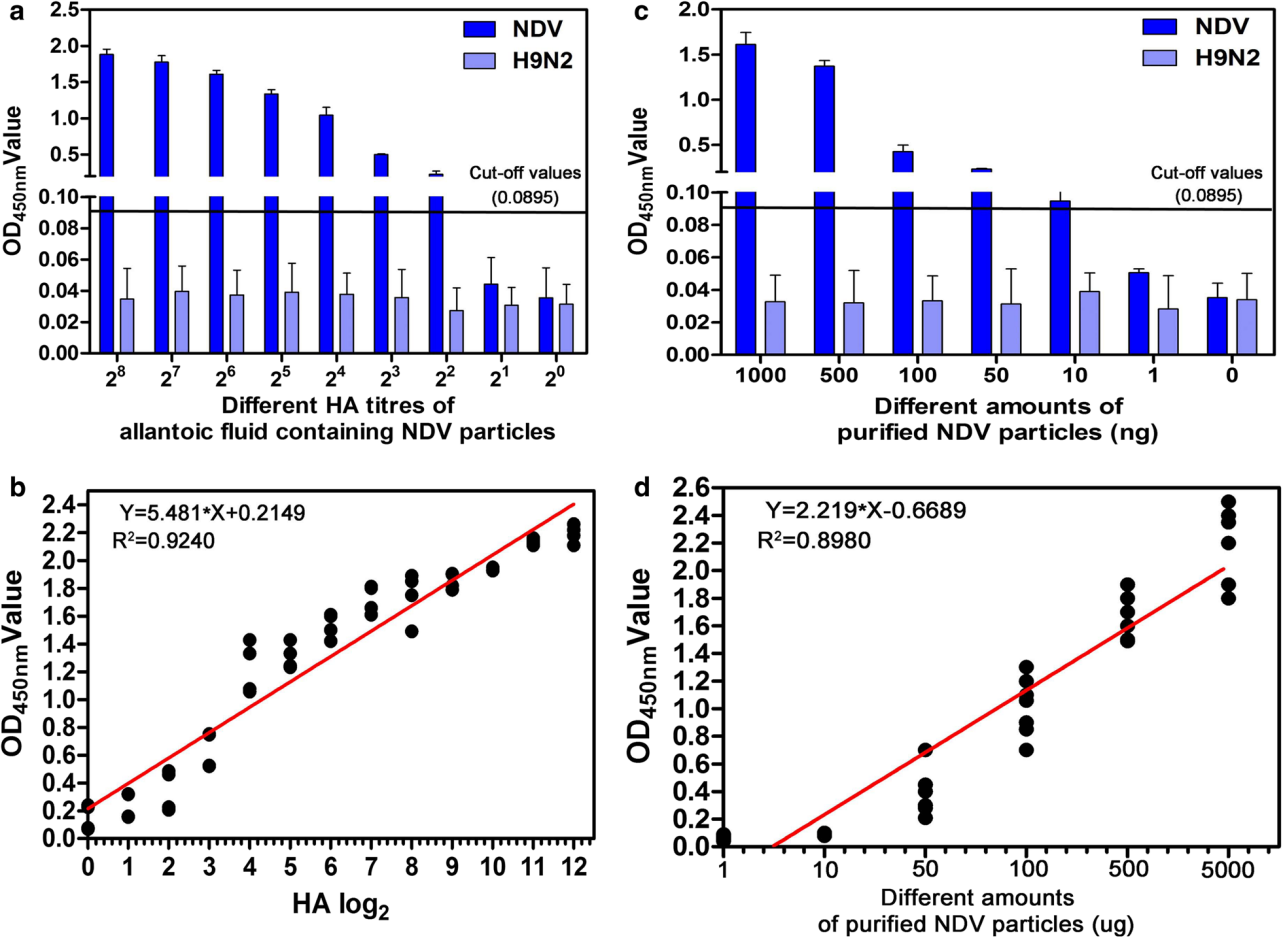
Determination of the detection limit of NDV for the developed fenobody and RANbody-based sandwich ELISA. **a** Different HA titers (2^8^ to 2^0^) of allantoic fluid containing NDV were detected with the developed sandwich ELISA. **b** A correlation was established between the developed sandwich ELISA and HA titers by a linear regression. **c** Different amounts of purified NDV particles were detected with the developed sandwich ELISA. **d** A correlation was established between the developed sandwich ELISA and amounts of purified NDV particles by linear regression

To determine the detection limit of the developed sandwich ELISA, different HA titers of allantoic fluid containing NDV were tested. The results show that the OD_450nm_ value was above 0.0895 when the titer of NDV in the allantoic fluid was 2^2^ (Fig. 5a). A close correlation (R^2^ = 0.9240) was found between the developed sandwich ELISA and HA titers (*P* < 0.0001) by a linear regression analysis (Fig. 5b). In addition, different amounts of purified NDV particles were also tested using the assay, which revealed that the OD_450nm_ value was above 0.0895 when the amount of purified NDV particles was 10 ng (Fig. 5c). A close correlation (R^2^ = 0.8980) was also found between the developed sandwich ELISA and different amounts of purified NDV particles (P < 0.001) (Fig. 5d).

**Fig. 6:**
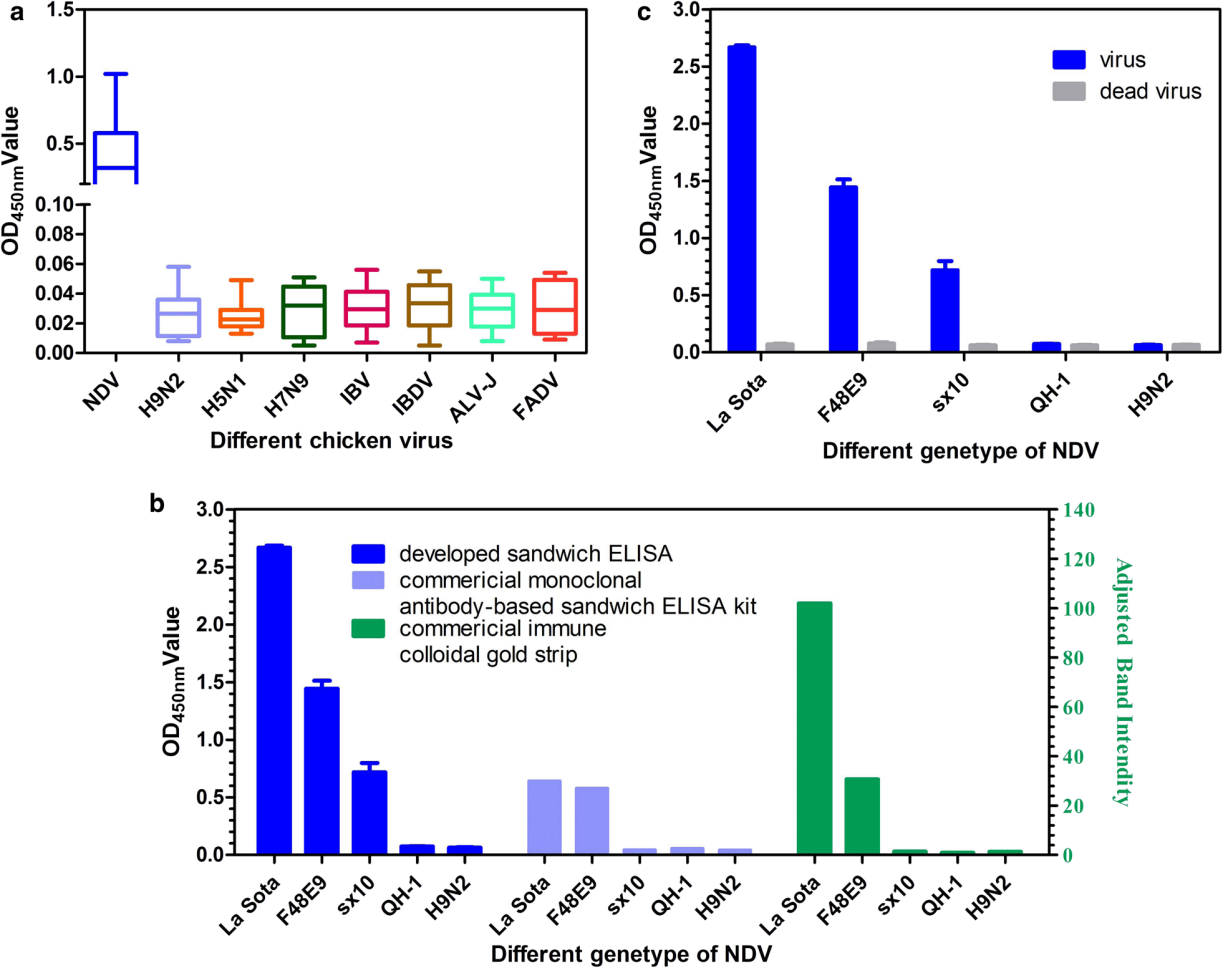
Analysis of the specificity of the developed fenobody and RANbody-based sandwich ELISA and the assay’s ability to detect the different NDV isolates or dead virus. **a** Using the developed sandwich ELISA to detect other chicken viruses, including H9N2 AIV, H5N1 AIV, H7N9 AIV, IBV, IBDV, ALV-J, and FADV. **b** Different NDV isolates detected with the developed sandwich ELISA, commercial monoclonal antibody-based sandwich ELISA, and the commercial immune colloidal gold strip. **c** Using the developed sandwich ELISA to detect the inactivated NDV strains

To evaluate the specificity of the sandwich ELISA, the other poultry viruses, H9N2, H5N1, H7N9, IBV, IBDV, FADV and ALV-J, were found to be negative with OD_450nm_ values from 0 to 0.058 (Fig. 6a).

In addition, different NDV isolates, Class II virulent strains F48E9 and sx10 and Class I strain QH-1, were detected using the developed sandwich ELISA. The results demonstrate that the developed sandwich ELISA can detect different Class II strains (LaSota, F48E9, and sx10) but not the Class I strain (QH-1) (Fig. 6b). However, when the four strains were detected using both the commercial monoclonal antibody-based sandwich ELISA and the immune colloidal gold strip, the results showed that both commercial assays can only detect the NDV LaSota and F48E9 strains but not sx10 and QH-1 (Fig. 6b). This finding indicates that the developed sandwich ELISA has higher sensitivity than the other two commercial assays. Moreover, the results show that the different inactivated NDV strains cannot be detected by the developed sandwich ELISA (OD_450nm_ values from 0 to 0.058), indicating that the assay only detects live NDV but cannot create false positive results for inactive NDV in the samples (Fig. 6c).

### Detection of NDV in chicken tissue samples with the developed sandwich ELISA

**Fig. 7:**
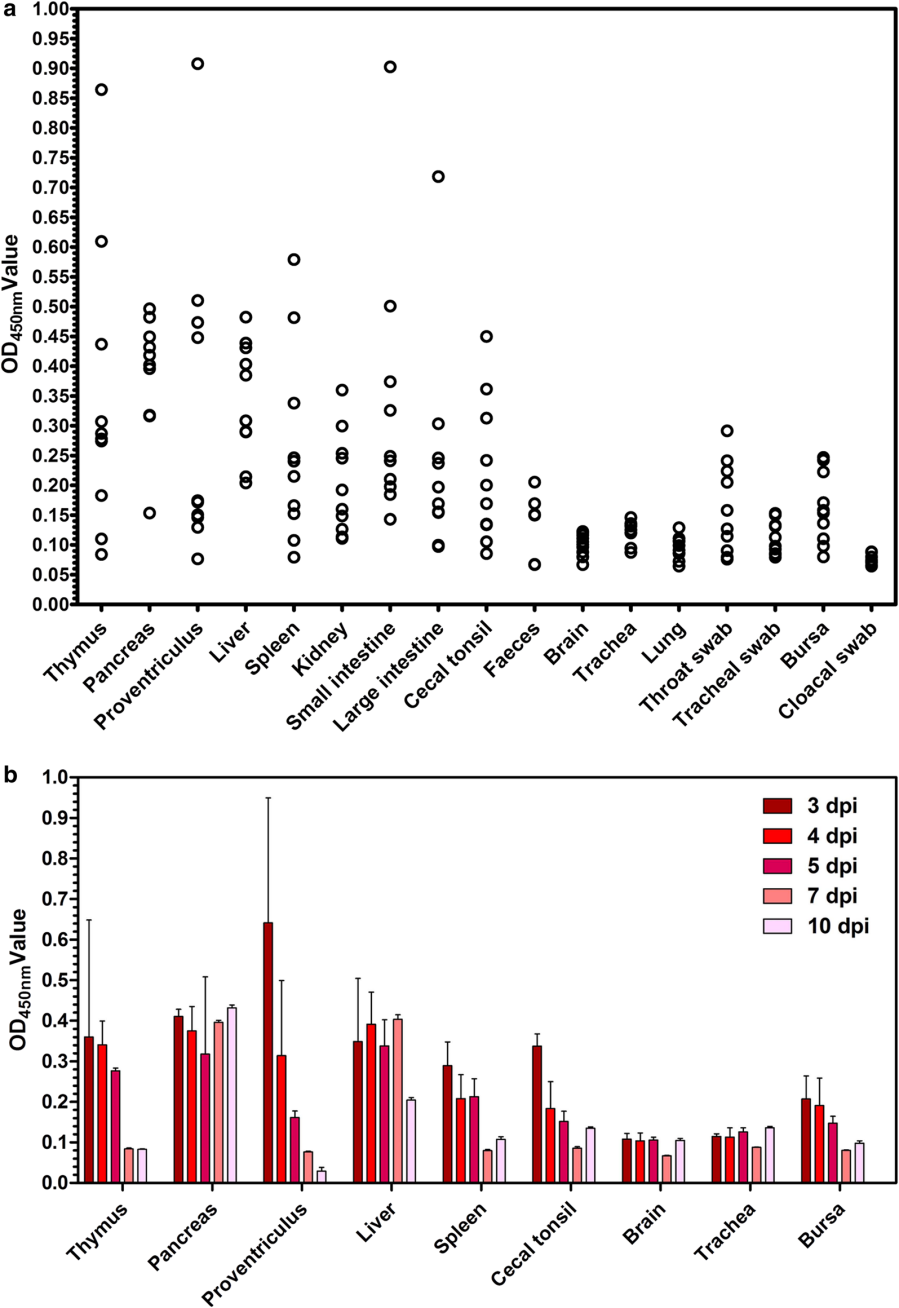
Detection of different tissue samples from the chickens infected with NDV using the developed fenobody and RANbody-based sandwich ELISA. **a** The distributions of OD_450nm_ values of the developed sandwich ELISA for detecting NDV from the different tissue samples. **b** Detection of NDV in the different dpi tissue samples from the challenged chickens. The tissue samples were collected at 3, 4, 5, 7 and 10 dpi from the infected chickens by necropsy

After testing the different tissue samples from the infected chickens, the results show that the developed sandwich ELISA can detect NDV in all samples, which includes thymus, proventriculus, small intestine, feces, throat swab, bursa, and other tissues (Fig. 7a). In addition, the amount of NDV in the chickens necropsied at 3, 4, and 5 dpi was higher than that in 7–10 dpi chickens (Fig. 7b).

### Agreements between developed sandwich ELISA with the commercial monoclonal antibody-based sandwich ELISA kit and with the commercial immune colloidal gold strip.

After testing 170 samples from the infected chickens using the developed sandwich ELISA, the commercial monoclonal antibody-based sandwich ELISA kit, and the commercial immune colloidal gold strip, the positive rates of each assay were 77.6% (132/170), 37.6% (64/170), and 9.4% (16/170), respectively (Table 6). The results of the developed sandwich ELISA and commercial monoclonal antibody-based sandwich ELISA kit coincided in 577 of the 707 samples with an agreement rate of 81.6% for both challenged and clinical chicken samples (Table 6). Similarly, the results of the developed sandwich ELISA and commercial immune colloidal gold strip agreed in 497 of the 707 samples with a rate of 70.3% (Table 6). Statistical analysis suggests that the developed sandwich ELISA has moderate consistency with the commercial monoclonal antibody-based sandwich ELISA (Kappa value = 0.523) and less consistency with the commercial immune colloidal gold strip (Kappa value = 0.146) (Table 6).

**Table 6:** Comparisons of the developed fenobody and RANbody-based sandwich ELISA with a commercial monoclonal antibody-based sandwich ELISA kit and a commercial immune colloidal gold strip by detecting challenged and clinical chicken tissue samples

Samples	Developed sandwich ELISA	Number	Commericial monoclonal antibody-based sandwich ELISA		Agreement (%)	Kappa value	Commericial immune colloidal gold strip		Agreement (%)	Kappa value
			+	−			+	−		
Challenged chicken	+	132	64	68	80.0	0.523	16	116	65.9	0.146
	−	208	0	208			0	208		
Clinical chicken	+	105	43	62	83.1		11	94	74.4	
	−	262	0	262			0	262		

The 340 samples from chickens contained 170 samples inoculated with NDV and 170 inoculated with PBS (from 3, 4, 5, 7, and 10 days post inoculation)

In addition, 237 positive samples detected by the developed sandwich ELISA were used to inoculate the SPF chicken embryos, then the allantoic fluid samples were detected with the HA test. The results show that 234 samples were positive, and the agreement rate of the two assays was 98.7%. Statistical analysis reveals that the developed sandwich ELISA has a high level of consistency with the HA test (Kappa value = 0.991). No significant difference was found between the fenobody and RANbody-based sandwich ELISA and HA tests (Kappa value was > 0.4).

## Discussion

To develop an effective sandwich ELISA, the pair of selected antibodies (capture and detection antibodies) is key in determining the sensitivity and specificity of the assay [38]. To date, traditional antibodies are universally used to develop sandwich ELISAs [8, 9, 39]. Yet, procedures of preparation, purification and labelling of traditional antibodies are complicated and costly, which is a heavy burden for the development of commercial sandwich ELISA kits [9, 25, 40]. In addition, the double-antibody sandwich ELISA is not extensively used for clinically detection of antigens and diagnosis of disease due to its high cost [41]. In the present study, the fenobody and RANbody derived from the nanobodies were first used to develop the sandwich ELISA for detecting the viral particles. For the assay, the fenobody was employed as the capture antibody and produced by expression with a bacterial system. The RANbody was employed as the detection antibody and produced by secretory expression with HEK293T cells. Compared to commercial sandwich ELISA kits produced with conventional antibodies, the productions of fenobody and RANbody are simpler and much less expensive. This is because the procedure to produce the fenobody only uses an *E. coli* system and RANbody production does not require purification or reporter-labelling. What’s more, no secondary antibody is required for detection. Compared with the conventional sandwich ELISA, designing the fenobody and RANbody-based sandwich ELISA follows a simplified production flowsheet, which reduces costs and operation time. More importantly, when nanobodies are available against other antigens, the method can be quickly applied to others.

The limited binding affinity of some nanobodies to antigens makes nanobodies inadequately rendered in bioanalytical applications that require high sensitivity [42, 43]. To date, oligomerisation of nanobodies has been mostly used to improve the binding affinity of nanobodies [43]. For instance, a previous study reported that fenobodies (displaying nanobodies on ferritin) are highly effective (more than 70-fold) for improving the binding affinity of nanobodies. They documented that the bacterial ferritin is a spherical protein that self-assembles nanocage from 24 subunits. The ferritin was genetically engineered by inserting nanobodies sequence at the C-terminus, thereby replacing the ε-helix of the *P. furiosus* ferritin subunit. Then, the ferritin fused nanobody (fenobody) can self-assemble into a nanocage and more nanobodies are displayed on the surface of nanocage [24, 44]. Then, the fenobody showed higher affinity to antigen compared with the traditional nanobody. In the presented study, the nanobody display platform was also used to reveal the NDV-specific nanobodies. Compared with the traditional nanobody against NDV, the fenobodies showed a higher binding affinity to NDV particles (LaSota strain), further indicating that fenobody is an easy and promising approach to improve the binding affinity of nanobody.

Newcastle disease causes a severe economic loss in the poultry industry worldwide due to the high costs of vaccinations and diagnostic laboratory investigations [45]. Among the assays for diagnosis and surveillance of the disease, the HA test, virus isolation by inoculating chicken embryo, sandwich ELISA, and immune colloidal gold strip are the most commonly methods used for detecting NDV particles in different samples [19, 46]. Generally, these assays have some shortcomings, including cumbersome operation, lengthy operation time, high cost of production, and low sensitivity for widespread use. In the present study, a sandwich ELISA for detecting NDV particles based on fenobodies and RANbodies against NDV was successfully developed. The assay exhibits high sensitivity, strong specificity, good reproducibility, and a high agreement rate with the HA test. In addition, the detection results of the sandwich ELISA have a close linear relationship with the HA titers and concentration of NDV particles, indicating that the amount of NDV particles in the samples can be determined by the sandwich ELISA. Compared with the previous assays for detecting NDV particles, the developed sandwich ELISA offers a simple method with low-cost production, high throughput, and rapid detection times. Therefore, we believe that the developed sandwich ELISA can be universally used to detect NDV particles in different samples, and the assay kit can be easily produced and implemented in the poultry industry.

To develop the high-sensitive sandwich ELISA platform, nanobodies against antigens were employed as important reagents. However, the screening procedures of the VHH genes from the immunized camel are complex and time-consuming and must be repeated many times to obtain the best nanobodies. In addition, it is difficult to attain expression of the fenobody in soluble form, which is also time-consuming and more laborious. Therefore, in the future works, different molecular techniques will be needed to minimize, or even inhibit, these disadvantages.

## Conclusion

In the present study, 13 NDV-specific nanobodies binding NDV particles were screened from an immunized Bactrian camel. Then, 5 fenobodies and RANbodies derived from the nanobody were separately produced. Based on these fenobodies and RANbodies, a sandwich ELISA using NDV-fenobody-4 and NDV-RANbody-49 as capture and detection reagents, respectively, was successfully developed for detecting NDV. The developed sandwich ELISA exhibits high sensitivity, good specificity, low cost, and simple commercial production and can be universally used to detect NDV in different samples. Overall, we believe that the fenobody and RANbody-based sandwich ELISA platform provides a versatile and scalable platform for rapidly developing the assay to detect antigens.

## Supplementary information

**Additional file 1: Fig. S1.** Evaluate the positive rate of the VHH library by colony PCR.** Fig. S2.** Purification of fenobodies by Capto Core 700 chromatography. The chromatogram showed fenobodies purification with Capto Core 700 in flow-through mode.** Table S1** Primer pairs for amplifying the VHH gene from the cDNA, identifying the positive clones by bacterial PCR, and amplifying fusion genes of fenobody. The underlying sequences in the primers were restriction sites for subsequently constructing recombinant plasmids.

## Data Availability

All data generated or analyzed during this study are included in the article.
