# Supplementary material for: Fenobody and RANbody-based sandwich enzyme-linked immunosorbent assay to detect Newcastle disease virus
Source: J Nanobiotechnology. 2020 Mar 14;18:44. doi: 10.1186/s12951-020-00598-2 (PMC7071587; doi:10.1186/s12951-020-00598-2)
Supplement: Supplementary file 1 — Additional file 1: Fig. S1. Evaluate the positive rate of the VHH library by colony PCR. Fig. S2. Purification of fenobodies by Capto Core 700 chromatography. The chromatogram showed fenobodies purification with Capto Core 700 in flow-through mode. Table S1 Primer pairs for amplifying the VHH gene from the cDNA, identifying the positive clones by bacterial PCR, and amplifying fusion genes of fenobody. The underlying sequences in the primers were restriction sites for subsequently constructing recombinant plasmids. [file 12951_2020_598_MOESM1_ESM.docx]

**Fenobody and RANbody-Based Sandwich Enzyme-linked Immunosorbent Assay to Detect Newcastle Disease Virus**

Pinpin Ji, Jiahong Zhu, Xiaoxuan Li, Wenqi Fan, Qianqian Liu, Kun Wang, Jiakai Zhao, Yani Sun, Baoyuan Liu, En-Min Zhou^*^ and Qin Zhao^*^

Department of Preventive Veterinary Medicine, College of Veterinary Medicine, Northwest A&F University；Scientific Observing and Experimental Station of Veterinary Pharmacology and Diagnostic Technology, Ministry of Agriculture, Yangling, Shaanxi, 712100, China.

**Table S1 Primer pairs for amplifying the VHH gene from the cDNA, identifying the positive clones by bacterial PCR, and amplifying fusion genes of fenobody. The underlying sequences in the primers were restriction sites for subsequently constructing recombinant plasmids.**

| **Primer names** | **Sequences (5’-3’)** |
| --- | --- |
| CALL001 | GTCCTGGCTGCTCTTCTACAAGG |
| CALL002 | GGTACGTGCTGTTGAACTGTTCC |
| VHH-FOR (*Pst* I) | CAGGTGCAGCTGCAGGAGTCTGGGGGAGR |
| VHH-REV (*Not* I) | CTAGTGCGGCCGCTGAGGAGACGGTGACCTGGGT |
| MP57 | TTATGCTTCCGGCTCGTATG |
| GIII | CCACAGACAGCCCTCATAG |
| NDV-2/4/24/30/49-FeNb –F (*Nde* I) | CCGCATATGATGCTGAGCGAACGCATGCTGA |
| NDV-2/4/24/30/49-Ferritin-R | ACTGCCTCCACCGCCACTGCCTCCACCGCCACTGCCTCCACCGCCGTCCTTGGCAAATTTCAGTTT |
| NDV-2/4/24/30/49-Nb-F | AAACTGAAATTTGCCAAGGACGGCGGTGGAGGCAGTGGCGGTGGAGGCAGTGGCGGTGGAGGCAGTCTGCAGGAGTCTGGGGGAGGCT |
| NDV-2/4/24/30/49-FeNb-R (*Bam*H I) | GACGGATCCTTAGGCGGCCGCTGAGGAGACGGTGACC |


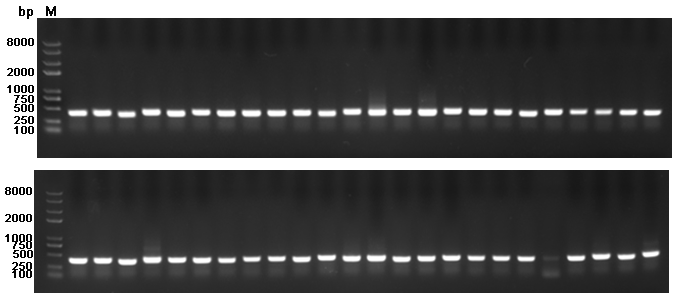


**Fig. S1 Evaluate the positive rate of the VHH library by colony PCR.**

**
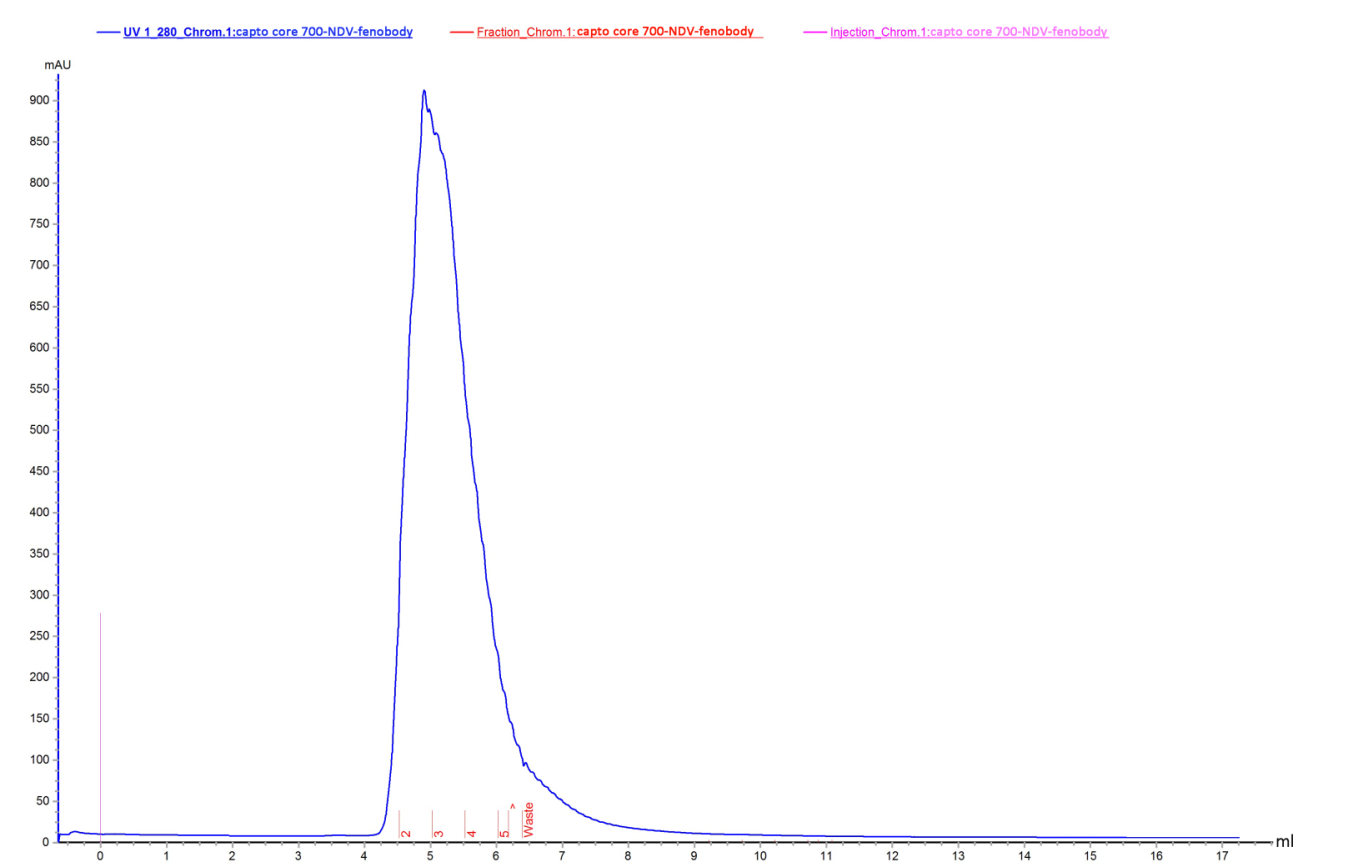
**

**Fig. S2 Purification of fenobodies by Capto Core 700 chromatography. The chromatogram showed fenobodies purification with Capto Core 700 in flow-through mode. Based on the manual instructions of the Capto Core 700 column, only the large biomolecules can be purified, indicating that the fenobody forming the particles. Purple line: positions of injection. Blue line: absorbance at 280 nm. Red line: positions of fractions.**
